# New Control Paradigms for Resources Saving: An Approach for Mobile Robots Navigation

**DOI:** 10.3390/s18010281

**Published:** 2018-01-18

**Authors:** Rafael Socas, Raquel Dormido, Sebastián Dormido

**Affiliations:** Departamento de Informática y Automática, Universidad Nacional de Educación a Distancia, Juan del Rosal 16, Madrid 28040, Spain; raquel@dia.uned.es (R.D.); sdormido@dia.uned.es (S.D.)

**Keywords:** event-based control, Networked Control Systems (NCS), resources efficiency, mobile robots, robot navigation

## Abstract

In this work, an event-based control scheme is presented. The proposed system has been developed to solve control problems appearing in the field of Networked Control Systems (NCS). Several models and methodologies have been proposed to measure different resources consumptions. The use of bandwidth, computational load and energy resources have been investigated. This analysis shows how the parameters of the system impacts on the resources efficiency. Moreover, the proposed system has been compared with its equivalent discrete-time solution. In the experiments, an application of NCS for mobile robots navigation has been set up and its resource usage efficiency has been analysed.

## 1. Introduction

In recent years, Networked Control Systems (NCS) have been gaining importance in the control community [1]. NCS are distributed architectures composed of controllers, sensors that can obtain information from the environment, actuators for acting on them, and a communication network that connects all the elements to achieve a common goal. Therefore, NCS is a field which includes different disciplines such as control theory, communications, software engineering and computer science. Typical applications where these control systems are being used are: space or terrestrial explorations, factory automation, remote diagnostics and troubleshooting, hazardous environments, experimental facilities, mobile robots, multi-vehicles networks, aircraft, manufacturing plant monitoring, nursing homes or hospitals, tele-robotics, tele-operation, etc.

The elements of an NCS system are called the agents. These elements use a communication network to exchange the information between them. Depending on the application where the NCS is used, this network can be deployed using wireline or wireless technology. These communication networks use digital technology to transmit the information which has constraints in delays and limited bandwidth. The information packaging and the constraints due to the limited resources of the network produce undesirable effects such as packet losses, variable delays and signal quantization issues among others. These effects may disturb the stability and performance of the system [2].

Therefore, reducing the traffic in the network is a critical aspect. If the number of packets is decreased can be guaranteed a predictable bandwidth, and at the same time, the analysis of the delays of the network is simplified [3]. As a conclusion, an important issue in the design of these control systems is to implement protocols for transmitting the sensor signals, the state of the system and the control information in a more effective way.

Some researchers have investigated the timing issues in NCS [4]. In traditional approaches, the controllers are used under the assumption of perfect communication, and then, the Maximum Allowable Transfer Interval (MATI) between two subsequent message transmissions that ensures closed loop stability under a network protocol is determined. Try Once Discard (TOD) and Round Robin (RR) are protocols implemented based on this philosophy. On the other hand, the MATI protocol is often deployed in a centralized way, therefore, it is not practical for systems of large-scale.

Other proposals have achieved an important reduction of network resources usage without a significant loss of performance. Two approaches have been raised: Model-Based Networked Control Systems (MBNCS) and Event-Based Control (EBC). The basics of the MBNCS have been developed in [5,6], and they have been considered in networks of coupled systems in [7] using periodic communication. Another approach to deal with this problem has been built on an event-based feedback scheme in NCS [8,9,10,11]. In event-based control systems, the agent information is broadcast only when some measures of the state error cross a specified level (the event threshold). This control scheme is decentralized in the sense that an agent can broadcast its state using the local information and in an asynchronous way.

In an EBC system, the impact of noise in the sensors increases the number of events and therefore a degradation of the performance. If the disturbance is known or can be modeled, the controller can be properly set to reduce its effects. However, in most applications it is difficult either to estimate the noise level or to have a reliable model. In these cases, it becomes a hard work to tune the controller in a proper way. In [12,13] these problems have been investigated where a new control scheme has been proposed which are dynamically adjusted depending on the conditions of the environment. In both proposals, the algorithms work with an estimation of the noise previously calculated. In [14], the estimation of the noise and the tune of the controller is made in real time.

The EBC strategies have been widely used to control dynamical processes while decreasing considerably the number of packets that the sensors have to send to the controller over the network. In [15] the events based on state errors have been investigated. In [16,17,18], similar proposals have been analysed to apply in networked interconnected systems. In these works, the use of a zero-order hold (ZOH) in the controller is a common feature. In [19], an event-triggering in networked systems with probabilistic sensor and actuator fault has been investigated to reduce the computation load. An overview on sampled-data-based event-triggered control and filtering for networked systems has been presented in [20]. In this research, a deep investigation of the sampled data-based event-triggered scheme has been made. In general, the event-based control architectures can be a good solution for the systems with limited resources and they could be a more efficient control scheme than the classical ones [21,22].

In this paper, a new event-based control architecture based on a simple event-based control scheme for NCS environments is presented. Making use of the control strategy implemented a full analysis of different resource consumption is carried out. The use of bandwidth, computational load and energy resources are analysed. Several methods and methodologies to measure the efficiency in the resources consumption are also proposed. The main contributions of this work are the models development of resource consumption and their parametrization. Finally, the ideas presented in this work are applied to an NCS mobile robots system to solve the navigation problem.

The paper is organized as follows. Section 2 presents an overview of the event-based control. In Section 3, the principles of sampling criteria are described. In Section 4, the proposed control strategy is presented. Section 5 shows the resource usage in the proposed system. Section 6 presents the experimental results. Finally, the conclusions and future work are discussed in Section 7.

## 2. Event-Based Control Overview

The event-based control has motivated the interest of the control community in the last few years, multiple control architectures and new applications have been propossed based on these ideas. In [23], an event-driven sampling method called the area-triggered method has been proposed. In this scheme, sensor data are sent only when the integral of the differences between the current sensor value and the last transmitted one is greater than a given threshold. The proposed system reduces the data transmission rate and also improves the estimation performance in comparison with the conventional time-driven technique. In [24], a greenhouse climate is controlled by an event-based control system. The system is based on a network of wireless sensors to control the low frequency dynamics of the environment. In this case, the control actions are calculated by considering the events that produce the external disturbances. The proposed system increases the actuators life and allows cost savings by minimizing the wear while maintaining a good performance. In [25], an event-based sampling according to a constant energy of sampling error is investigated. The defined criterion is suitable for applications where the energy of the sampling error should be bounded (e.g., in greenhouse climate monitoring and control or in building automation). Finally, in [26], a fault isolation filter to apply on discrete-time networked control systems based on a particular form of the Kalman filter is proposed. The scheme makes an efficient use of the resources with a good estimation of failures and its effect on the performance. The sampled-data-based event-triggered control schemes is another emerging event-based control technique. The reliable control design for networked control system under event-triggered scheme is investigated in [19]. The key idea of this work is that only the newly sampled sensor measurements that violate specified triggering condition will be transmitted to the controller. The main advantage of this approach is that the proposed event-triggered scheme only needs a supervision of the system state in discrete instants and there is no need to retrofit the existing system. Finally, in [20], an overview and a deep investigation on sampled-data-based event-triggered control and filtering for networked systems has been done. Compared with some existing event-triggered and self-triggered schemes, a sampled-data-based event-triggered scheme can ensure a positive minimum inter-event time and make it possible to jointly design suitable feedback controllers and event-triggered threshold parameters.

In event-based control systems, information is exchanged between the elements (controller, sensors and actuators) depending on the state of the system [27]. When the system variables exceed a certain level an event is generated in the system and the control actions are executed. This means that the activity of the controller and the use of resources to communicate the different elements are restricted to the time intervals in which a control action must inevitably be taken to guarantee the system specifications.

**Figure 1:**
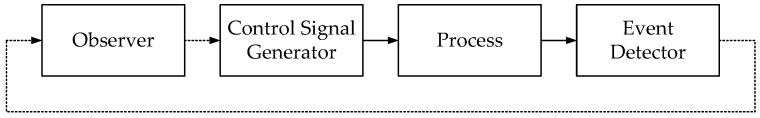
Basic scheme of an event-based control system. Solid arrows represents continuous signal and dashed arrows represents event-based signals.

In Figure 1, the basic scheme of an event-based control strategy is presented [28,29].

The control scheme is composed of an event detector, an observer, and a control signal generator. The event detector generates an output signal when an event occurs, it happens when the error signal crosses a threshold. When an events occurs, the observer is updated and it passes the information to the control signal generator. With this information, the control signal generator generates the input signal to control the process. An important aspect of this strategy is that the observer and the control signal generator works in open loop between events.

This control architecture combines feedback and feedforward strategies. When an event is generated there is a feedback action. On the other hand, the feedforward actions happen when the actuators are driven by the control signal generator in open loop between events.

## 3. Event-Based Sampling Schemes

**Figure 2:**
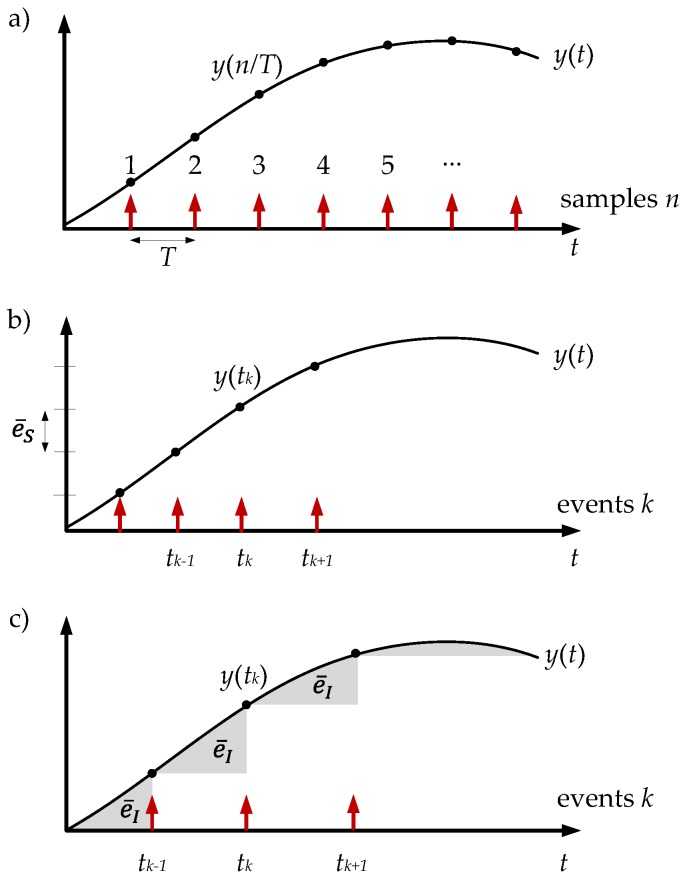
Sampling strategies: (**a**) periodic sampling; (**b**) send-on-delta; and (**c**) integral criterion.

Different sampling criteria have been proposed in the event-based control schemes [27]. In the event-based sampling methods, the system acts only when the variables of the plant are in transient state. In the steady state the system does not act and some resources can be saved. On the other hand, in discrete time schemes the sampling is executed periodically (with the period $T=1/f_{s}$ where $f_{s}$ is the sampling frequency) and it does not depend on the state of the system (Figure 2a).

Send-on-delta and integral criterion are the most widely used techniques in event-based control schemes. In the following sections, these methods will be defined and their effectiveness will be discussed.

### 3.1. Send-On-Delta

The send-on-delta sampling algorithm is the most natural signal-dependent strategy; in the literature, it is also known as level-crossing or deadbands sampling. In the send-on-delta technique, the sensors do not broadcast a new message if the signal remains within a certain level of confidence ${\bar{e}}_{S}$ (resolution) (Figure 2b). The sampling criterion is defined as

(1) $$|y\left(t\right)-y\left(t_{k}\right)|\geq {\bar{e}}_{S}$$

The ratio of events that the send-on-delta algorithm generates can not be calculated in a general way, but its average value $N_{S}$ may be estimated by the following expression [30,31]:

(2) $$N_{S}=\frac{1}{\bar{\Delta t}}$$

where $\bar{\Delta t}$ is the mean period between events considering the analysis interval $\left(t_{0},t_{n}\right)$

In the send-on-delta algorithm, an event occurs when

(3) $$|y\left(t_{k}\right)-y\left(t_{k-1}\right)|={\bar{e}}_{S}\;,\;k=1,2,\ldots ,n$$

and the interval time between the events k−1 and *k* is expressed as

(4) $$\Delta t_{k}=t_{k}-t_{k-1}=\frac{{\bar{e}}_{S}}{\bar{|\dot{y}\left(t_{k}\right)|}}$$

where $\bar{|\dot{y}\left(t_{k}\right)|}$ is defined as

(5) $$\bar{|\dot{y}\left(t_{k}\right)|}=\frac{1}{\Delta t_{k}}\int_{t_{k}}^{t_{k+1}}\left|\dot{y}\left(t\right)\right|dt$$

at this point, the mean period between events $\bar{\Delta t}$ can be defined as

(6) $$\bar{\Delta t}=\frac{\sum_{k=1}^{n}\Delta t_{k}}{n}$$

in [31], the following relationship has been proven

(7) $$\bar{\Delta t}=\frac{{\bar{e}}_{S}}{\bar{|\dot{y}\left(t\right)|}}$$

where $\bar{|\dot{y}\left(t\right)|}$ was defined as

(8) $$\bar{|\dot{y}\left(t\right)|}=\frac{1}{t_{n}-t_{0}}\int_{t_{0}}^{t_{n}}\left|\dot{y}\left(t\right)\right|dt$$

taking Equations (2) and (7) into account, the mean rate of events $N_{S}$ can be written as

(9) $$N_{S}=\frac{\bar{|\dot{y}\left(t\right)|}}{{\bar{e}}_{S}}$$

In this case, the ratio of events $N_{S}$ in the system depends on two parameters:

- the resolution ${\bar{e}}_{S}$ of the sampling (the event threshold); and
- the mean of the absolute value of the first time-derivative $\bar{|\dot{y}\left(t\right)|}$ during the analysis interval.

As presented in Equation (9), the message rate in the send-on-delta strategy is a trade off between the resolution ${\bar{e}}_{S}$ and the average slope of the signal y(t).

### 3.2. Integral Criterion

There are some reasons to apply the event-based integral criterion in control systems. This method has a high efficiency for sampling burst signals. Likewise, this technique is a good solution in applications where a critical problem of sampling process is the accuracy of approximation of a continuous-time signal by a sequence of discrete-time samples. In this algorithm, the sampling criterion (Figure 2c) is defined by Equation (10), where ${\bar{e}}_{I}$ is the resolution of the method:

(10) $$\int_{t_{k}}^{t}\left|y\left(t\right)-y\left(t_{k}\right)\right|dt\geq {\bar{e}}_{I}$$

then, the mean rate of events $N_{I}$ can be estimated as follows [32]:

The system generates an event when the following condition is satisfied

(11) $$\int_{t_{k}}^{t_{k+1}}\left|y\left(t\right)-y\left(t_{k}\right)\right|dt={\bar{e}}_{I}$$

taking into account the time interval between the events *k* and k+1 ($\Delta t_{k}=t_{k+1}-t_{k}$), in [32], it has been demonstrated that the mean period between events considering the interval $\left(t_{0},t_{n}\right)$ is

(12) $$\bar{\Delta t}=\frac{\sum_{k=1}^{n}\Delta t_{k}}{n}=\frac{\sqrt{2{\bar{e}}_{I}}}{\bar{\sqrt{|\dot{y}\left(t\right)|}}}$$

where $\bar{\sqrt{|\dot{y}\left(t\right)|}}$ is defined by

(13) $$\bar{\sqrt{|\dot{y}\left(t\right)|}}=\frac{1}{t_{n}-t_{0}}\int_{t_{0}}^{t_{n}}\sqrt{|\dot{y}\left(t\right)|}dt$$

Finally, the mean rate of events $N_{I}$ based on the integral criterion strategy can be expressed as:

(14) $$N_{I}=\frac{1}{\bar{\Delta t}}=\frac{\bar{\sqrt{|\dot{y}\left(t\right)|}}}{\sqrt{2{\bar{e}}_{I}}}$$

Taking Equation (14) into account, the mean rate of events depends on the mean of the square root of the signal derivative absolute value and the resolution ${\bar{e}}_{I}$ used in the algorithm

### 3.3. Effectiveness of Event-Based Sampling

To study how effective the presented sampling algorithms are, they will be compared with periodic sampling strategies. In the send-on-delta algorithm, the mean rate of events $N_{S}$ was estimated in Equation (9). If the periodic sampling algorithm is considered, the sampling period *T* can be obtained by Equation (15) where the accuracy of the algorithm is ${\bar{e}}_{P}$

(15) $$T=\frac{{\bar{e}}_{P}}{|\dot{y}{\left(t\right)|}_{max}}$$

Now, the ratio of samples $N_{P}$ can be calculated as

(16) $$N_{P}=\frac{1}{T}=\frac{|\dot{y}{\left(t\right)|}_{max}}{{\bar{e}}_{P}}$$

If the send-on-delta is compared with the periodic sampling considering the same resolution for both methods (${\bar{e}}_{S}={\bar{e}}_{P}$), the following equation is obtained

(17) $$\frac{N_{S}}{N_{P}}=\frac{\bar{|\dot{y}\left(t\right)|}}{|\dot{y}{\left(t\right)|}_{max}}$$

In this case, $N_{S}=N_{P}$ if y(t) is a linear signal, for the rest of the continuous-time signal $N_{S}<N_{P}$ is fulfilled. The last expression implies that the send-on-delta is more efficient than the periodic sampling algorithm.

Taking into account the integral criterion, the number of samples in the periodic sampling strategy is given by (see [32]):

(18) $$N_{P}=\frac{\sqrt{2{\bar{e}}_{P}}}{\sqrt{|\dot{y}{\left(t\right)|}_{max}}}$$

If both methods (event-based and periodic) have the same accuracy (${\bar{e}}_{I}={\bar{e}}_{P}$), the following equation is obtained from Equations (14) and (18):

(19) $$\frac{N_{I}}{N_{P}}=\frac{\bar{\sqrt{|\dot{y}\left(t\right)|}}}{\sqrt{|\dot{y}{\left(t\right)|}_{max}}}$$

which shows that for the integral criterion the event-based strategy is more efficient than the periodic sampling $N_{I}<N_{P}$ in a general way.

It can be concluded that the event-based sampling algorithms are more efficient than the periodic strategies. It means that the event-based solution generates less events than the periodic sampling scheme.

## 4. NCS Control Architectures

In this section, two NCS schemes are discussed. First, the classical discrete-time architecture is presented, and then the event-based solution proposed in this work is investigated in detail. In these NCS, the agents (the controller and the remote node) are connected by a wireless network.

**Figure 3:**
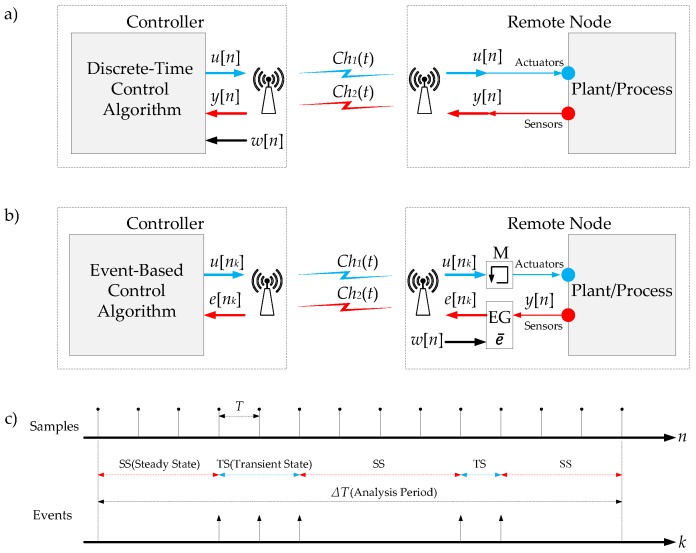
NCS control schemes: (**a**) discrete-time; (**b**) event-based; and (**c**) time diagrams. The samples represent the activity of the discrete-time system; for the event-based solution, the activity is represented by the events.

The basic scheme of a discrete-time NCS is presented in Figure 3a. The signals u[n] (control signal) and y[n] (sensor signal) are sampled with the frequency $f_{s}$. The signal u[n] is sent to remote node over the communication channel $Ch_{1}\left(t\right)$. In the remote node, this information is used to act over the actuators. The signal y[n] is sent to the controller over the $Ch_{2}\left(t\right)$. Finally, the controller calculates u[n] considering the reference signal w[n] and y[n]. Therefore, this architecture exchanges information over the communication channels every period of time defined by $T=1/f_{s}$. When the plant is in a steady state, it is not necessary to interchange information between the elements of the system because the plant does not need new control actions. However, in this scheme, the communication channels $Ch_{1}\left(t\right)$ and $Ch_{2}\left(t\right)$, the controller, the actuators and the sensors are busy every period of time *T*.

The proposed event-based NCS is presented in Figure 3b. As mentioned above, the proposed system follows the basic principles of an event-based control system, being the evaluation of the consumption of resources the main objective of this work. The system is composed of a controller, an event generator (EG) and a memory block (M). In the EG the signals, y[n] and w[n] are compared. If the difference between these signals crosses the event threshold $\bar{e}$, the system generates an event *k* and the signal $e[n_{k}]$ (error signal) is sent to the controller. Different methods can be applied to produce events, in [21], a review of these methodologies is presented. In a general way, the event threshold $\bar{e}$ is defined as a constant value. This parameter can be defined as a function of the noise in the sensors or as a function of other relevant variables of the process to generate the events in a more accuracy way. In [12,14], these ideas have been explored. Therefore, the event threshold has to be set as a trade off between the accuracy of the system and the number of events. The communication channel $Ch_{2}\left(t\right)$ is used to send the signal $e[n_{k}]$ to the controller. In this case, the RF channel is occupied only when the EG generates events. On the other hand, when the signals w[n] and y[n] are very similar, the plant/process is in a steady state. In this case, no events occur in the system and the channel $Ch_{2}\left(t\right)$ is free. When the signal $u[n_{k}]$ arrives in the remote node it is stored in the memory *M*. Therefore, every time the system generates an event, the memory is updated with a new value. In the periods of time between events, the information stored in the memory is used to control the remote node. Besides, when an event is generated in the system, the controller receives the signal $e[n_{k}]$ and it calculates the signal $u[n_{k}]$. Afterwards, this control signal is sent to the remote node via $Ch_{1}\left(t\right)$. As result of this, the communication resources $Ch_{1}\left(t\right)$ and $Ch_{2}\left(t\right)$ are used only when the system is generating events.

In general, the communication channels $Ch_{1}\left(t\right)$ and $Ch_{2}\left(t\right)$ use Industrial, Scientific and Medical (ISM) bands which are not exclusive and many agents may be using them at the same time. When other devices are using the same channel, the interferences, the packet dropouts and an excessive delay in the network could affect the performance of the control system. To avoid these effects, a free channel in the radio link between the remote node and the controller has to be selected. For example, if the 2.4 GHz band is used, there are around 126 available channels so there is a high probability of finding interference-free channels in the wireless network.

As a conclusion, the proposed event-based solution has three main advantages:

1. The RF channels are busy only when the system generates events, see Figure 3c.
2. The controller does not need to compute control signals when the plant is in the steady state.
3. The system is protected from Zeno phenomenons. In the proposed control scheme the events are generated in the system only at certain instants of time (Figure 3c). This behaviour sets a minimum time interval between events defined by $1/f_{s}$. This is a typical mechanism to avoid the Zeno phenomenons in the event-based control systems [33].

In a practical way, to compare the responses of both NCS architectures (discrete-time and event-based), some conditions must be imposed:

- The clocks of the systems are synchronized (Figure 3c). This implies that the events and the samples in the system are generated at the same instant of time.
- The accuracy of both systems has to be the same. In this case, the event threshold has to be set up considering the sampling frequency of the discrete-time system and the sampling criterion in the event-based one.

## 5. Resources Usage

To measure the performance of a control system, the Integral Absolute Error IAE is applied. This criterion is widely used in continuous-time and discrete-time control systems. The IAE calculates the difference between the output of the system (y(t) for continuous-time or y[n] for discrete-time) and the reference signal (w(t) or w[n]) by the following equations:

(20) $$\begin{array}{c} IAE=\int_{t_{0}}^{t}\left|w\left(t\right)-y\left(t\right)\right|dt\\ IAE=\sum_{n_{0}}^{n}\left|w\left[n\right]-y\left[n\right]\right| \end{array}$$

Therefore, the analysed system has a good performance if the IAE is small.

Another way to evaluate the performance of a control system is by the Integral Absolute Error compared to Periodic loop IAEP. This criterion is mainly used in event-based control systems [34,35]. This indicator compares the output of the event-based system $y_{eve}\left[n\right]$ with the output of its equivalent discrete-time system y[n], as presented in Equation (21).

(21) $$IAEP=\sum_{n_{0}}^{n}\left|y_{eve}\left[n\right]-y\left[n\right]\right|$$

Although the IAEP is a good criterion to measure the performance of an event-based control system, in practice, many researchers [36,37,38,39,40] use the ratio of events $N_{N}$. This parameter calculates the activity of an event-based control system versus an equivalent discrete-time control system, the ration of events is given by:

(22) $$N_{N}=\frac{N_{eve}}{N_{per}}$$

where $N_{eve}$ is the number of events in the analysed system and $N_{per}$ the number of samples in the equivalent discrete-time system. In this case, the number of events can be defined as $N_{eve}=N_{S}\Delta T$ for the send-on-delta algorithm and $N_{eve}=N_{I}\Delta T$ for the integral criterion, where ΔT is the analysis period. In the same way, the number of samples is defined as $N_{per}=N_{P}\Delta T$ (see Section 3). In this context, if $N_{N}<1$ the event-based system has less activity than the equivalent discrete-time system and consequently, it uses less resources. As was previously analysed, the event-based sampling criteria generate less events than the periodic sampling techniques using the same resolution in both methods. It means that $N_{P}\geq N_{EVE}$ (where $N_{EVE}=N_{S}$ if the send-on-delta is used or $N_{EVE}=N_{I}$ if the integral criterion is used (see Equations (17) and (19)). If the analysis period ΔT is taken into account, it can be written $N_{eve}=N_{EVE}\Delta T$ and $N_{per}=N_{P}\Delta T$, then the ratio of events is given as:

(23) $$N_{N}=\frac{N_{eve}}{N_{per}}=\frac{N_{EVE}}{N_{P}}$$

where $0\leq N_{N}\leq 1$.

Using the event efficiency $\eta_{N}$, this can be expressed as:

(24) $$\eta_{N}=\left(1-N_{N}\right)100$$

In a general way, if the resolutions in the sampling methods are the same, the activity of the event-based control systems is more efficient than the discrete-time solutions ($0\%\geq \eta_{N}\geq 100\%$).

In this work, to analyse the resources usage efficiency, the ratio $N_{R}$ is defined as follows

(25) $$N_{R}=\frac{{\left(R\right)}_{eve}}{{\left(R\right)}_{dis}}$$

where *R* indicates which resource is analysed, ${\left(R\right)}_{eve}$ is the usage of the resource *R* in the event-based control system and ${\left(R\right)}_{dis}$ denotes the usage of the same resource in the equivalent discrete-time control system. In the same way, the efficiency in the usage of the resource *R* can be expressed as

(26) $$\eta_{R}=\left(1-N_{R}\right)100$$

In the following sections, the usage efficiency of the resources of the control system such as bandwidth, computational load and energy are investigated.

### 5.1. Bandwidth Usage

**Figure 4:**
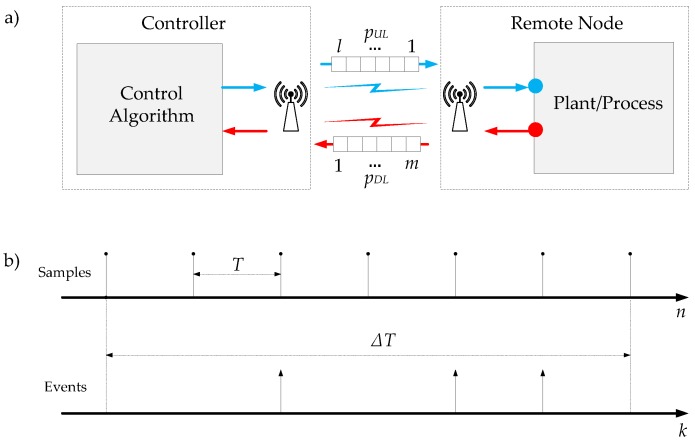
Bandwidth utilization model: (**a**) control architecture; and (**b**) time diagrams for discrete-time and event-based schemes.

The model depicted in Figure 4a has been used to analyse the bandwidth usage.

The information in the uplink direction is $p_{UL}=l$ bits and in the downlink is $p_{DL}=m$ bits. The analysis period ΔT is considered. In this interval time, the discrete-time system generates $N_{per}$ samples and the event-based system $N_{eve}$ events. Then, the bandwidth usage, defined as the number of bits transmitted per second, can be written for the discrete-time system by

(27) $${\left(BW_{UL}\right)}_{dis}=\frac{lN_{per}}{\Delta T};{\left(BW_{DL}\right)}_{dis}=\frac{mN_{per}}{\Delta T}$$

and for the event-based solution is given by

(28) $${\left(BW_{UL}\right)}_{eve}=\frac{lN_{eve}}{\Delta T};{\left(BW_{DL}\right)}_{eve}=\frac{mN_{eve}}{\Delta T}$$

where $BW_{UL}$ and $BW_{DL}$ represent the bandwidth usage in the uplink and in the downlink direction, respectively.

The bandwidth usage ratio for the uplink $N_{BW_{UL}}$ and for the downlink $N_{BW_{DL}}$ can be expressed by the following equations

(29) $$N_{BW_{UL}}=\frac{{\left(BW_{UL}\right)}_{eve}}{{\left(BW_{UL}\right)}_{dis}}=\frac{N_{eve}}{N_{per}}=N_{N}$$

(30) $$N_{BW_{DL}}=\frac{{\left(BW_{DL}\right)}_{eve}}{{\left(BW_{DL}\right)}_{dis}}=\frac{N_{eve}}{N_{per}}=N_{N}$$

considering $N_{BW_{UL}}=N_{BW_{DL}}$, the bandwidth usage ratio for both directions $N_{BW}$ can be defined as

(31) $$N_{BW}=\frac{N_{eve}}{N_{per}}=N_{N}$$

and the bandwidth usage efficiency is given by

(32) $$\eta_{BW}=\eta_{N}$$

As Equation (32) shows, the bandwidth usage efficiency is the same as the event efficiency.

### 5.2. Computational Load Reduction

**Figure 5:**
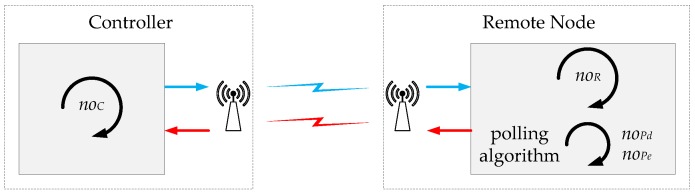
Computational resources model.

To obtain a model of the computational load in the analysed control systems, the scheme depicted in Figure 5 has been used.

The control algorithm is composed of two elements: the algorithm in the controller and the algorithm in the remote node. In the remote node, the algorithm is divided in two blocks, the control algorithm and the polling algorithm. The polling block is used to get the measures from the sensors and in the event-based solution it also generates the events. In the controller, $no_{C}$ operations are executed each time the algorithm is run. On the other hand, in the remote node, $no_{R}$ operations are executed in the control algorithm either $no_{Pd}$ when the discrete-time solution is used or $no_{Pe}$ when the event-based technique is selected.

Taking into account the previous assumptions, the computational load in the discrete-time system ${\left(CL\right)}_{dis}$ and in the event-based control system ${\left(CL\right)}_{eve}$ considering the analysis period ΔT can be expressed by

(33) $${\left(CL\right)}_{dis}=\frac{\left(no_{C}+no_{R}+no_{Pd}\right)N_{per}}{\Delta T}$$

(34) $${\left(CL\right)}_{eve}=\frac{\left(no_{C}+no_{R}\right)N_{eve}+no_{Pe}N_{per}}{\Delta T}$$

then, the computational load ratio $N_{CL}$ is given by

(35) $$N_{CL}=\frac{{\left(CL\right)}_{eve}}{{\left(CL\right)}_{dis}}=\frac{\left(no_{C}+no_{R}\right)N_{N}+no_{Pe}}{no_{C}+no_{R}+no_{Pd}}$$

and the computational load efficiency can be written as

(36) $$\eta_{CL}=\left(1-N_{CL}\right)100$$

In general, if $N_{N}$ increases, the efficiency $\eta_{CL}$ decreases. If the computational load of the polling algorithms is smaller than the control algorithms ($no_{Pd}<<\left(no_{C}+no_{R}\right)$ and $no_{Pe}<<\left(no_{C}+no_{R}\right)$), $N_{CL}\approx N_{N}$ and $\eta_{CL}\approx \eta_{N}$. On the other hand, when the load of the polling algorithms increases, the computational load ratio ($N_{CL}\to 1$) and the event-based control system does not have efficiency in computational load $\eta_{CL}\to 0\%$. As a conclusion, the computational load efficiency of the system has a high dependence on the computational load of the polling algorithms.

From a practical point of view, to evaluate the computational burden of the system a simple procedure has to be followed. First, it is necessary to distinguish which part of the algorithm is executed periodically and which one is executed eventually when the events occur in the system. Then, the parameters $no_{C}$, $no_{R}$, $no_{Pd}$ and $no_{Pe}$ can be obtained doing a high level analysis of the control algorithm. In Section 6.3.2 and in Appendix A, this procedure has been applied in the application used as practical example in this work.

### 5.3. Energy Consumption

**Figure 6:**
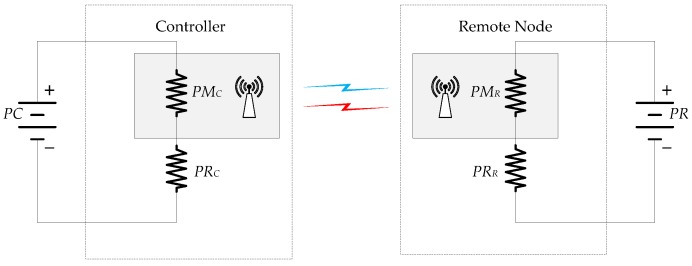
Energy model for the controller and for the remote node.

In this section, the energy efficiency of the presented event-based control system is investigated. In Figure 6, the simplified energy model of the system is depicted.

The electric power in the controller can be obtained adding up the power that the modem needs to transmit and receive the information $PM_{C}$ and the power in the rest of the system of the controller $PR_{C}$. When the discrete-time system is considered, the power of the controller is given by

(37) $$PC_{dis}=PM_{C}N_{per}+PM_{C}\left(\frac{1-\beta }{\beta }\right)$$

where β is the power ratio defined by

(38) $$\beta =\frac{PM_{C}}{PM_{C}+PR_{C}}$$

Using the same argument, the power of the controller when the event-based architecture is used can be written as

(39) $$PC_{dis}=PM_{C}N_{eve}+PM_{C}\left(\frac{1-\beta }{\beta }\right)$$

Taking into account the analysis period ΔT, the energy usage in the discrete-time controller ${\left(EC\right)}_{dis}$ and for the event-based controller ${\left(EC\right)}_{eve}$ are given by the following equations

(40) $${\left(EC\right)}_{dis}=\left(PM_{C}N_{per}+PM_{C}\left(\frac{1-\beta }{\beta }\right)\right)\Delta T$$

(41) $${\left(EC\right)}_{eve}=\left(PM_{C}N_{eve}+PM_{C}\left(\frac{1-\beta }{\beta }\right)\right)\Delta T$$

and the energy ratio in the controller $N_{EC}$ can be expressed as

(42) $$N_{EC}=\frac{{\left(EC\right)}_{eve}}{{\left(EC\right)}_{dis}}=\frac{N_{eve}\beta +\left(1-\beta \right)}{N_{per}\beta +\left(1-\beta \right)}$$

and the energy efficiency in the controller can be written as

(43) $$\eta_{EC}=\left(1-N_{EC}\right)100$$

Using the same reasoning in the remote node, the energy usage in the discrete-time implementation ${\left(ER\right)}_{dis}$ and in the event-based solution ${\left(ER\right)}_{eve}$ can be written as

(44) $${\left(ER\right)}_{dis}=\left(PM_{R}N_{per}+PM_{R}\left(\frac{1-\gamma }{\gamma }\right)\right)\Delta T$$

(45) $${\left(ER\right)}_{eve}=\left(PM_{C}N_{eve}+PM_{C}\left(\frac{1-\gamma }{\gamma }\right)\right)\Delta T$$

where $\gamma =\frac{PM_{R}}{PM_{R}+PR_{R}}$. Then, the energy ratio in the remote system is defined by

(46) $$N_{ER}=\frac{{\left(ER\right)}_{eve}}{{\left(ER\right)}_{dis}}=\frac{N_{eve}\gamma +\left(1-\gamma \right)}{N_{per}\gamma +\left(1-\gamma \right)}$$

and the energy efficiency in the remote node can be written as

(47) $$\eta_{ER}=\left(1-N_{ER}\right)100$$

If the power of the modem in the controller is high (β→1), the efficiency in energy usage in the proposed event-based system is the event efficiency $N_{EC}\to N_{N}$ and $\eta_{EC}\to \eta_{N}$. On the other hand, when the power of this device is low (β→0), the system is not energy efficient, $N_{EC}\to 1$ and $\eta_{EC}\to 0\%$. Similar conclusions can be obtained in the remote node considering the parameter γ. In conclusion, the power of the modems determines the energy efficiency of the proposed event-based control scheme.

## 6. Experimental Results

**Figure 7:**
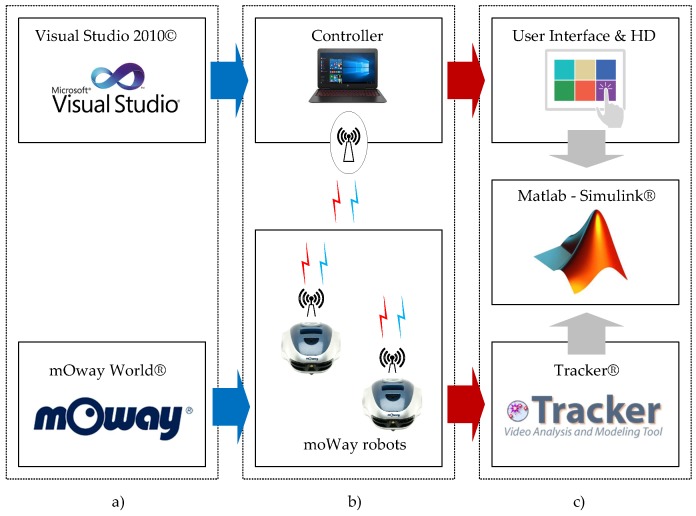
Laboratory for the experiments: (**a**) development module; (**b**) mobile robots environment; and (**c**) analysing tools.

To check the ideas presented in this work, a test laboratory to investigate wireless control systems has been developed (Figure 7). In this platform, the controller has been implemented in a laptop and the remote nodes are the mobile robots. For this purpose, mOway mobile robots [41] have been used. Using this platform, an analysis of the behaviour of the event-based control schemes presented in this paper is carried out as well as a comparison with their equivalent discrete-time implementation.

The control algorithms have been programmed in C++ for the controller and in the mOway World environment for the robots (Figure 7a). A radio link interface is used to communicate the controller and the robots (Figure 7b). Finally, other applications such as the Tracker, the Matlab/Simulink and some scripts in the controller can be used to analyse the experimental results (Figure 7c).

**Figure 8:**
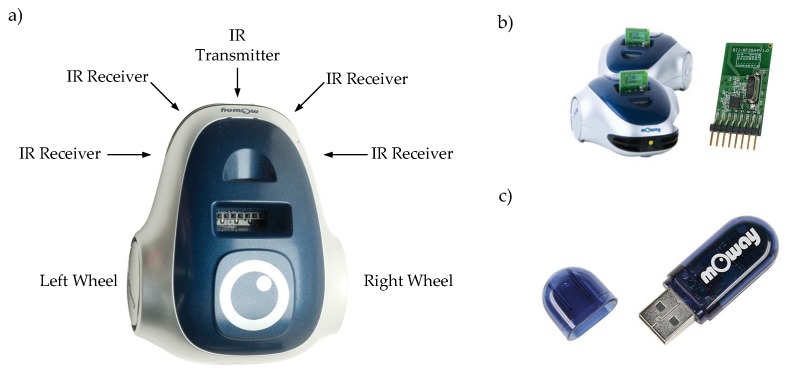
Robot platform: (**a**) components and sensor distribution; (**b**) robot wireless interface; and (**c**) PC wireless interface.

The structure and components of the robots are depicted in Figure 8. The robots have four infra-red obstacle sensors with a maximum range of 3 cm and a sensor to measure the battery level (Figure 8a). The wireless communication system (robot RF interface (Figure 8b), and PC RF interface (Figure 8c)) works in the worldwide ISM frequency band at 2.400–2.4835 GHz and uses GFSK modulation. In the system 126 channels can be configured, the rate for each channel is 2 Mbps. The angular speeds of the wheels can be varied from 0 to 10.9 rad/s and their geometrical parameters L=6.6 cm (distance between wheels) and r=1.6 cm (radius of the wheels).

To analyse the event-based control architecture proposed in this work and its resources usage efficiency, some experiments have been set up. In these experiments robot navigation algorithms will be checked in the laboratory. In the following sections, the proposed navigation algorithms, their implementation in the system and the experimental results will be analysed in detail.

### 6.1. Navigation Algorithms

In navigation applications for mobile robots, the algorithms such as Go To Goal (GTG), Obstacle Avoidance (OA) and Wall Following (WF) are widely used [42,43,44,45,46]. In these applications, it is also critical to define a precise positioning mechanism to guarantee the convergence of the navigation algorithm [47,48].

**Figure 9:**
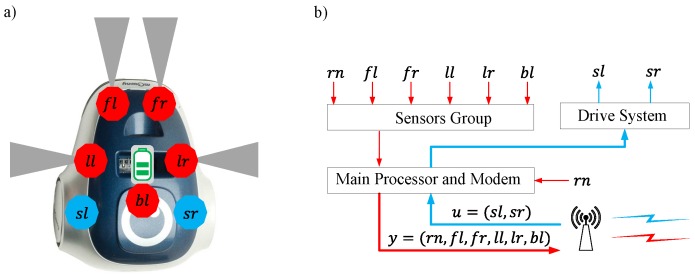
Sensors and actuators of the mOway robot: (**a**) position and orientation of the IR obstacle sensors (fl,fr,ll,lr), the battery sensor (bl), and the wheel actuators (sl,sr); and (**b**) flow diagram for the communication between sensors, actuators and the RF interface.

In this work, the behaviour and the resource usage of the OA and WF algorithms are investigated in the proposed event-based control architecture. To define the navigation algorithms, the position and the orientation of the obstacle sensors have to be taken into account (Figure 9a). The variables that contain the measurements of the sensors are defined by two indexes as follows: ll lateral left, fl front left, fr front right and lr lateral right. The parameter bl stores the battery level. In the actuators, the linear speeds of the wheels are sl speed left and sr speed right. Finally, the parameter rn (robot number) identifies the robot in the platform.

The sensor information y=(rn,ll,fl,fr,lr,bl) and the control signals u=(sl,sr) are sent and received by the RF interface, as shown in Figure 9b.

**Figure 10:**
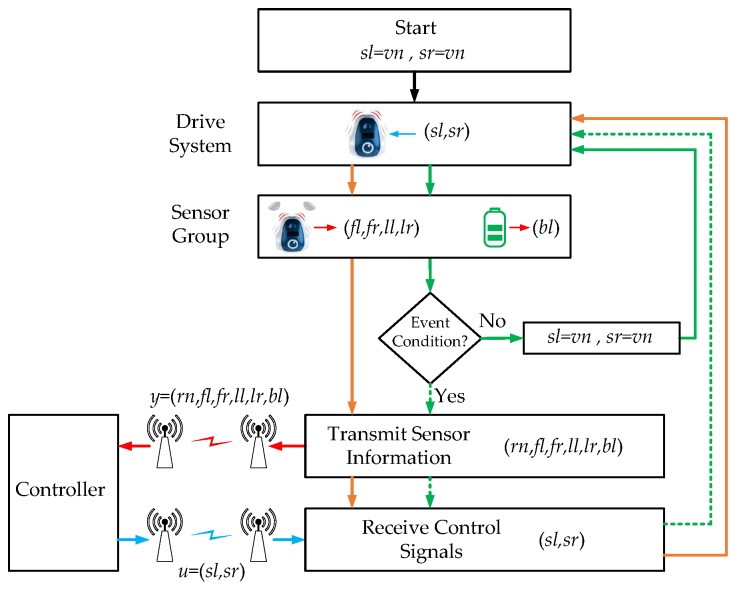
Robot control algorithm. The orange arrows represent the discrete-time solution and the green arrows the event-based implementation. The blocks with continuous arrows are executed each period of time *T* and the blocks with dotted arrows when an event is generated in the system.

The architecture of the algorithm in the robots is depicted in Figure 10.

The orange arrows represent the discrete-time implementation. The green ones are used for the event-based solution. The discrete-time algorithm works as follows:

1. The navigation speed $v_{n}$ is assigned to sl and sr.
2. The speeds sl and sr are applied to the actuators.
3. The sensors group gets the information from the sensors.
4. The sensor information *y* is sent to the controller.
5. The control signals *u* are received from the controller.
6. Finally, the speeds are applied to the robot wheels.

In the event-based solution, Steps 4 and 5 are executed only if the event condition is fulfilled. Furthermore, depending on which algorithm is executing (OA or WF) the event condition will be different. In the following subsections, these aspects and the controller algorithm will be defined in detail.

#### 6.1.1. Obstacles Avoidance Algorithm

In the event-based implementation of the OA algorithm, the event condition is given by

(48) $$\begin{array}{c} \mathrm{if}\;(\left(fl>{\bar{e}}_{OA}\right)\;\mathrm{OR}\;\left(fr>{\bar{e}}_{OA}\right)\;\mathrm{OR}\ldots \\ \ldots \left(ll>{\bar{e}}_{OA}\right)\;\mathrm{OR}\;\left(lr>{\bar{e}}_{OA}\right))\\ \left\{event=true\right\}\\ \mathrm{else}\;\{event=false\} \end{array}$$

where ${\bar{e}}_{OA}$ is the event threshold. As shown in Equation (48), if any of the obstacle sensors (fl,fr,ll,lr) exceeds the value of the event threshold (${\bar{e}}_{OA}$), an event is generated in the system and the robot sends the sensor information to the controller. The value of the event threshold determines the accuracy of the algorithm and the number of the events that the robot generates. If the threshold value is high, the number of events decreases at the same time as the accuracy of the algorithm does. On the other hand, there will be an inverse behaviour when the value of the event threshold decreases.

**Figure 11:**
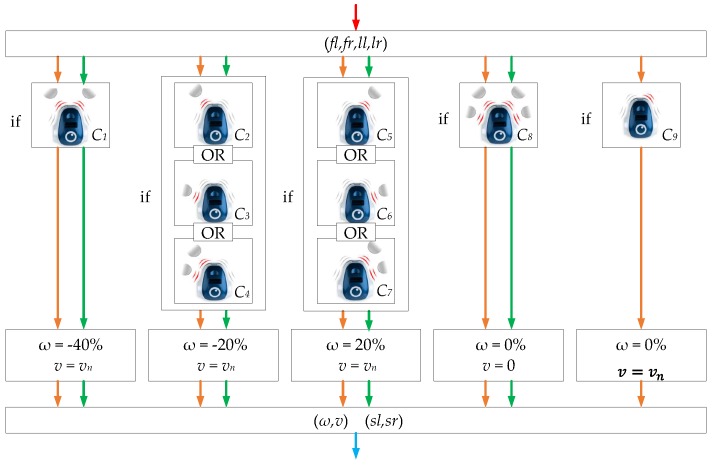
Obstacles avoidance algorithm in the controller. The blocks with orange arrows are executed in the discrete-time implementation and those with green arrows in the event-based one.

The algorithm in the controller is shown in Figure 11 and in Equation (49). In this case, the same algorithm is use for the two implementations (discrete-time and event-based).

(49) $$\begin{array}{c} \mathrm{if}\;\left(C_{1}\right)\;\left\{\omega =-40\%,v=v_{n}\right\}\\ \mathrm{else}\;\mathrm{if}\;\left(C_{2}\;\mathrm{OR}\;C_{3}\;\mathrm{OR}\;C_{4}\right)\;\left\{\omega =-20\%,v=v_{n}\right\}\\ \mathrm{else}\;\mathrm{if}\;\left(C_{5}\;\mathrm{OR}\;C_{6}\;\mathrm{OR}\;C_{7}\right)\;\left\{\omega =20\%,v=v_{n}\right\}\\ \mathrm{else}\;\mathrm{if}\;\left(C_{8}\right)\;\left\{\omega =0\%,v=0\right\}\\ \mathrm{else}\;\left(C_{9}\right)\;\left\{\omega =0\%,v=v_{n}\right\} \end{array}$$

where conditions $C_{1}$–$C_{9}$ are defined by Equations (50)–(58)

(50) $$\begin{array}{c} \mathrm{if}\;(ll=0\;\mathrm{AND}\;fl>0\;\mathrm{AND}\;fr>0\;\mathrm{AND}\;lr=0)\\ \left\{C_{1}=true\right\} \end{array}$$

(51) $$\begin{array}{c} \mathrm{if}\;(ll=0\;\mathrm{AND}\;fl>0\;\mathrm{AND}\;fr=0\;\mathrm{AND}\;lr=0)\\ \left\{C_{2}=true\right\} \end{array}$$

(52) $$\begin{array}{c} \mathrm{if}\;(ll>0\;\mathrm{AND}\;fl=0\;\mathrm{AND}\;fr=0\;\mathrm{AND}\;lr=0)\\ \left\{C_{3}=true\right\} \end{array}$$

(53) $$\begin{array}{c} \mathrm{if}\;(ll>0\;\mathrm{AND}\;fl>0\;\mathrm{AND}\;fr=0\;\mathrm{AND}\;lr=0)\\ \left\{C_{4}=true\right\} \end{array}$$

(54) $$\begin{array}{c} \mathrm{if}\;(ll=0\;\mathrm{AND}\;fl=0\;\mathrm{AND}\;fr>0\;\mathrm{AND}\;lr=0)\\ \left\{C_{5}=true\right\} \end{array}$$

(55) $$\begin{array}{c} \mathrm{if}\;(ll=0\;\mathrm{AND}\;fl=0\;\mathrm{AND}\;fr=0\;\mathrm{AND}\;lr>0)\\ \left\{C_{6}=true\right\} \end{array}$$

(56) $$\begin{array}{c} \mathrm{if}\;(ll=0\;\mathrm{AND}\;fl=0\;\mathrm{AND}\;fr>0\;\mathrm{AND}\;lr>0)\\ \left\{C_{7}=true\right\} \end{array}$$

(57) $$\begin{array}{c} \mathrm{if}\;(ll>0\;\mathrm{AND}\;fl>0\;\mathrm{AND}\;fr>0\;\mathrm{AND}\;lr>0)\\ \left\{C_{8}=true\right\} \end{array}$$

(58) $$\begin{array}{c} \mathrm{if}\;(ll=0\;\mathrm{AND}\;fl=0\;\mathrm{AND}\;fr=0\;\mathrm{AND}\;lr=0)\\ \left\{C_{9}=true\right\} \end{array}$$

In Figure 11, the orange arrows represent the discrete-time solution and the green arrows the proposed event-based control algorithm. This algorithm is parametrized by the linear speed *v* and the angular speed ω. Theses magnitudes are transformed into wheel speeds sl and sr by

(59) $$sl=v-\omega \frac{L}{2}\omega_{max}$$

(60) $$sr=v+\omega \frac{L}{2}\omega_{max}$$

where ω is expressed as a percentage of the angular speed and $\omega_{max}$ is the maximum angular speed of the robot. Notice that, in this control algorithm, condition $C_{9}$ is only executed in the discrete-time implementation.

As presented in Figure 11, the algorithm receives from the robot the sensor information (fl,fr,ll,lr). In the discrete-time implementation, this information is received every period of time $T=1/f_{s}$ and in the event-based solution when an event is generated in the robot. Depending on the values of the sensors (conditions $C_{1}$ to $C_{9}$), different control actions are taken. In this implementation, ω>0 represents a turn of the robot to the left, on the contrary ω<0 does the robot turns to the right. For example, if the right sensors detect an obstacle (the conditions $C_{5}$ OR $C_{6}$ OR $C_{7}$ is fulfilled) the robot must turn to the left. In this case, the control law is defined as ω=20% (turn to left) and $v=v_{n}$ (maintain constant linear velocity).

#### 6.1.2. Wall Following Algorithm

Two variants of the WF algorithm are usually implemented: clockwise (CW) or counter-clockwise (CCW) [45]. In this work, the second option has been selected. The event condition in the algorithm is defined by

(61) $$\begin{array}{c} \mathrm{if}\;(\left(fl>0\right)\;\mathrm{OR}\;\left(abs\left(ll-w\right)>{\bar{e}}_{WF}\right)\\ \left\{event=true\right\}\\ \mathrm{else}\;\{event=false\} \end{array}$$

where ${\bar{e}}_{WF}$ represents the event-threshold and the parameter *w* represents the target distance between the robot and the wall. As presented in Equation (61), if there is an obstacle in front of the robot (fl>0) or the distance from the robot to the wall exceeds the event threshold ($abs\left(ll-w\right)>{\bar{e}}_{WF}$), an event is generated in the robot.

The controller algorithm, which is the same in both architectures (discrete-time and event-based), is defined by

(62) $$\begin{array}{c} \mathrm{if}\;((fl>0)\\ \left\{sl=v_{n},sr=0\right\}\\ \mathrm{else}\;\{sl=\left(ll-w\right)v_{w}+v_{n},sr=v_{n}\} \end{array}$$

where $v_{w}$ represents the approach speed to the wall. In this case, the control law is the same for both implementations (discrete-time and event-based). In this algorithm, when there is an obstacle in front of the robot (fl>=0), the left wheel rotates with a constant speed ($sl=v_{n}$) and the right wheel stops (sr=0). In any other situation, the speed of the right wheel remains constant ($sr=v_{n}$) and the right wheel is modulated according to $v_{w}$ to maintain a constant distance *w* to the wall.

### 6.2. System Activity

In this work, two experiments have been set up, the OA algorithm has been checked in experiment 1 and the FW in experiment 2. In both experiments, the discrete-time architecture was implemented in robot number 1 (rn=1) and the event-based one in robot number 2 (rn=2).

The discrete-time system works with a sampling frequency of 10 Hz, and the event-based system uses the send-on-delta sampling method. The obstacles sensors have a maximum change rate (mcr) of 3 cm/s. To compare the efficiency of both systems (discrete-time and event-based), they must have the same accuracy. In other words, the precision of the discrete-time system ${\bar{e}}_{P}$ has to be the same as ${\bar{e}}_{S}$ (see Section 3.3). In this case, ${\bar{e}}_{P}=mcr/f_{s}$ and ${\bar{e}}_{S}$ is defined as ${\bar{e}}_{OA}$ for the OA algorithm and as ${\bar{e}}_{WF}$ for the WF algorithm. Taking into account the previous assumptions, the event thresholds were set up to ${\bar{e}}_{OA}={\bar{e}}_{WF}={\bar{e}}_{P}=mcr/f_{s}=0.3$ cm.

The parameters of the algorithms are presented in Table 1 and Table 2.

In the experiments, the responses of the systems were analysed during fifteen minutes (ΔT=15 min). To analyse the activity of the control scheme, the number of samples $N_{per}$ in the discrete-time system and the number of events $N_{eve}$ in the proposed architecture have been measured.

**Figure 12:**
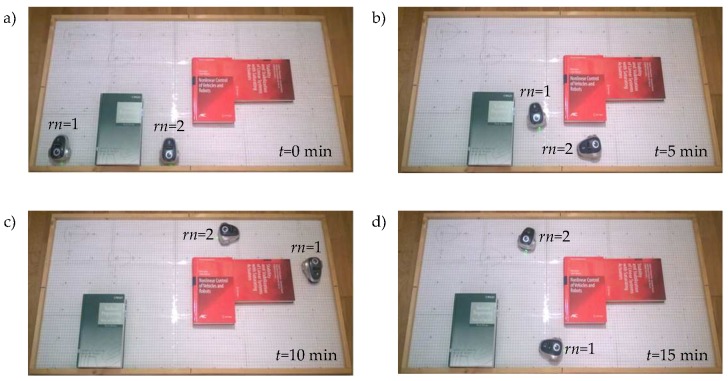
Snapshots of experiment 1. Positions of the robots at: (**a**) t=0 min; (**b**) t=5 min; (**c**) t=10 min; and (**d**) t=15 min.

**Figure 13:**
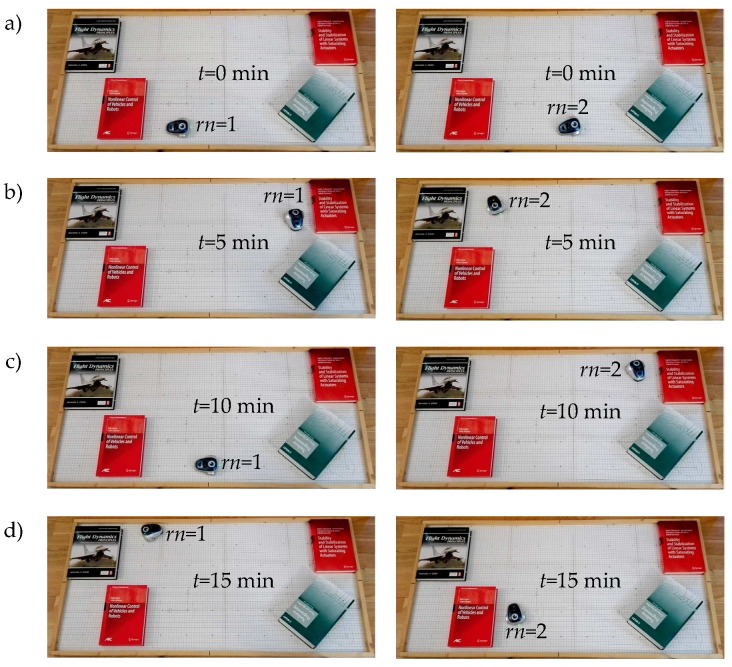
Snapshots of experiment 2. Positions of the robots at: (**a**) t=0 min; (**b**) t=5 min; (**c**) t=10 min; and (**d**) t=15 min.

In Figure 12 and Figure 13, some snapshots of the experiment are shown. In both experiments, the robots show a stable behaviour. The control scheme solve the navigation problem in the discrete-time solution rn=1 as in the event-based implementation rn=2.

**Figure 14:**
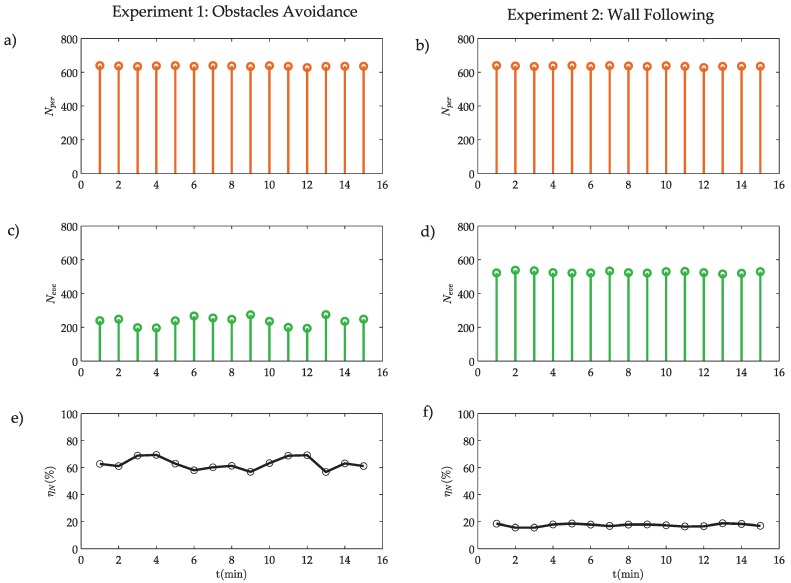
Activity of the systems: number of samples $N_{per}$ (**a**,**b**); number of events $N_{eve}$ (**c**,**d**); and event efficiency $\eta_{N}$ (**e**,**f**).

In Figure 14, the activity of the systems is presented.

In both experiments, the number of samples $N_{per}$ in the discrete-time architecture is the same (Figure 14a,b). On the other hand, the number of events $N_{eve}$ is always smaller than the number of samples $N_{per}$ in Equation (17) (Figure 14c,d), as demonstrated in Section 3.3. In this case, the event efficiency $\eta_{N}$ for the 15 min experiment is 63% for the OA and 17% for WF algorithm, as presented in Figure 14e,f and in Table 3.

### 6.3. Resource Efficiency

In this section, the RF bandwidth, the computational load and the energy consumption in both architectures have been analysed. At the same time, the experimental results are discussed.

#### 6.3.1. RF bandwidth

In each sensor and in each actuator, the transmitted information is a 8 bit code. Taking into account this assumption, the RF uplink (from controller to robot) uses 16 bits and the RF downlink (from robot to controller) needs 48 bits to send a packet of information (see Figure 10). Some additional bits also have to be included to manage the radio interface. In this case, the downlink is the critical link because it needs most of the bandwidth.

**Figure 15:**
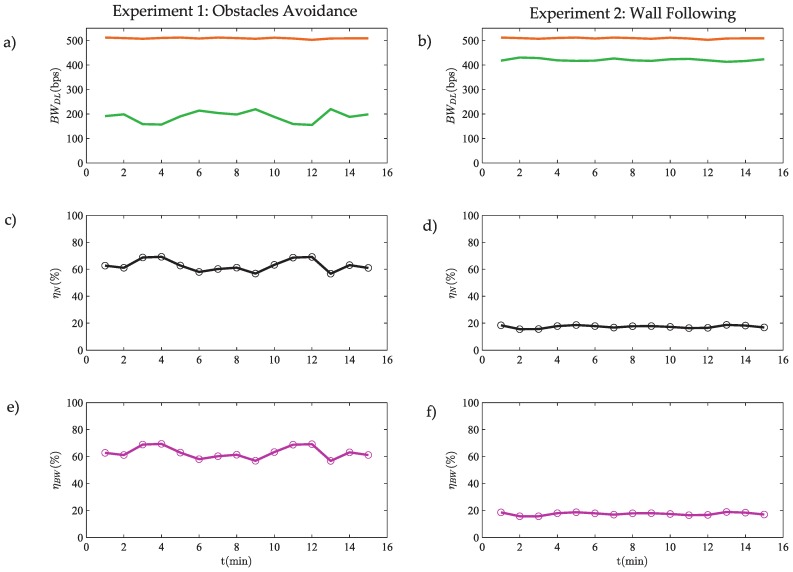
Bandwidth efficiency: (**a**,**b**) download bandwidth, where orange line represents the discrete-time architecture and the green line the event-based architecture; (**c**,**d**) event efficiency $\eta_{N}$; and (**e**,**f**) download bandwidth efficiency $\eta_{BW}$.

The downlink (DL) bandwidth for the two experiments is depicted in Figure 15a,b.

As demonstrated in Section 5.1, the efficiency in the bandwidth usage $\eta_{BW}$ (Figure 15e,f) is the same as the event efficiency $\eta_{N}$ (32) (Figure 15c,d). The average bandwidths for each architecture (${\left({\bar{BW}}_{DL}\right)}_{dis}$, ${\left({\bar{BW}}_{DL}\right)}_{eve}$), the bandwidth usage ratio $N_{BW}$ and the bandwidth usage efficiency $\eta_{BW}$ are presented in Table 4.

Taking into account these results, the bandwidth efficiency is 63% for the OA algorithm and 17% for the WF one.

#### 6.3.2. Computational Load

To estimate the computational load in both experiments, a high level analysis of the algorithms has been performed. In Table 5, the number of operations for each algorithm is presented.

The parameters $no_{C}$, $no_{R}$, $no_{Pd}$ and $no_{Pe}$ have been calculated taking into account the high level representation of the algorithms of the robots and the controllers (see Figure 5 and Appendix A). Considering Table 5 and the activity of the system ($N_{per}$ and $N_{eve}$), the computational load for each experiment can be calculated by Equations (33)–(35).

**Figure 16:**
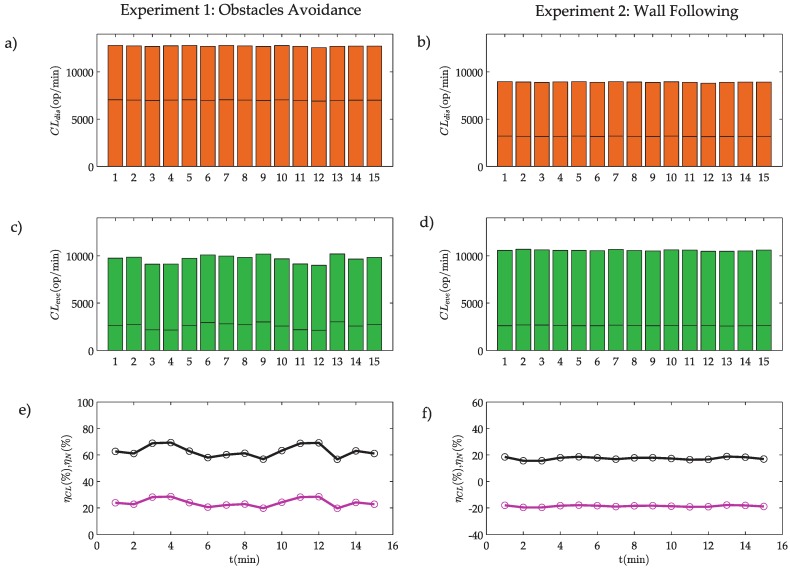
Computational load: (**a**,**b**) Computational load in the discrete-time implementation, where the bottom bars represent the controller and the top bars the robot; (**c**,**d**) computational load in the event-based implementation, where the bottom bars represent the controller and the top bars the robot; and (**e**,**f**) event efficiency $\eta_{N}$ (black line) and computational load efficiency $\eta_{CL}$ (pink line).

The results for experiments 1 and 2 are depicted in Figure 16.

In experiment 1, the computational load is lower in the event-based implementation than in the discrete-time one (Figure 16a,c). In this case, the computational load efficiency $\eta_{CL}$ reaches 24% (Figure 16e and Table 6). On the other hand, in experiment 2, the computational load in the event-based solution is higher than the discrete-time one (Figure 16b,d). In this case, the computational load efficiency is −19% (Figure 16f and Table 6).

As discussed in Section 5.2, the computational load has a high dependence on the ratio of events $N_{N}$ and the polling algorithms. In these experiments, the polling algorithms have a small computational load (see Table 5), but the ratio of events is very large especially in the WF algorithm (see Table 3). This is the main reason why the efficiency in WF algorithm is negative. Therefore, the proposed system does not present good results for computational load in the WF algorithm. In this case, the solution could be to modify the event threshold ${\bar{e}}_{WF}$ to improve the efficiency with the inconvenience of reducing the accuracy of the system.

#### 6.3.3. Energy Consumption

In these experiments, the controller has been implemented in a laptop with Windows OS. In this system, it is very complicated to measure the consumed energy by the controller and therefore this consumption has not been considered. On the other hand, in the mOway robot measuring this energy is easy by using the battery level sensor bl.

**Figure 17:**
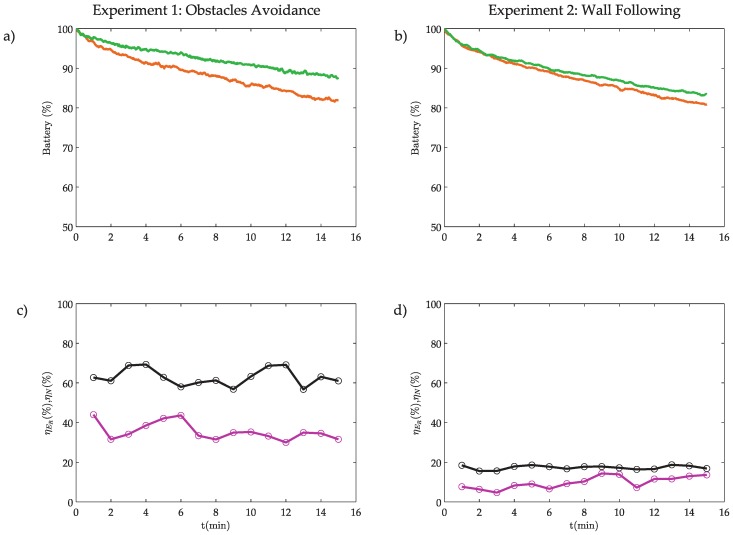
Energy consumption: (**a**,**b**) battery level, where the orange line represents the battery level in the discrete-time robot, and the green line in the event-based robot; and (**c**,**d**) event efficiency $\eta_{N}$ (black line) and robot energy efficiency $\eta_{CL}$ (pink line).

The results of the experiments are depicted in Figure 17.

As shown in Figure 17a,b, when both experiments end, the battery level in the proposed event-based architecture is higher than in the discrete-time implementation, which means that the proposed system uses less energy than the classical discrete-time solution. The energy used in the robot can be estimated in the discrete-time solution as

(63) $${\left(ER\right)}_{dis}=100\%-{\left(Battery\left(\%\right)\right)}_{dis}$$

and in the event-based architecture by

(64) $${\left(ER\right)}_{eve}=100\%-{\left(Battery\left(\%\right)\right)}_{eve}$$

where ${\left(Battery\left(\%\right)\right)}_{dis}$ denotes the level of battery measured in sensor bl for the discrete-time solution and ${\left(Battery\left(\%\right)\right)}_{eve}$ for the event-based one.

In these experiments, the energy ratio in the robot can be obtained directly by $N_{ER}=\frac{{\left(ER\right)}_{eve}}{{\left(ER\right)}_{dis}}$ and energy efficiency by $\eta_{ER}=\left(1-N_{ER}\right)100$. The results are presented in Figure 17c,d and Table 7.

In this case, the energy efficiency is 31% for experiment 1 and 14% for experiment 2. The energy consumed by the robot could be directly obtained using the measures of the sensor bl and this has allowed to calculate the energy efficiency directly. On the other hand, using the model developed in Section 5.3, the power ratio γ can be obtained. In these experiments γ<1%, this means that the modem of the robot consumes less than 1% of the robots energy. This value is extremely small because the modem has very little power (< 1 mW) and consequently a short range (less than 20 m).

As analysed previously, if the power of the modem increases, the efficiency also increases. In these examples, if the power of the modem is increased (e.g., 100 mW which implies a range of 1 km, γ=63%) the efficiency in the OA algorithm increases from 31% to 62% and in the WF algorithm from 14% to 17%.

## 7. Conclusions and Future Work

The event-based control architectures presented in this work can be an alternative to the classical discrete-time control systems. The features of these new control schemes help to manage the resources of the system under optimal conditions because they present high efficiency in the resource usage. In a general way, by using a sampling criterion such as the send-on-delta or the integral criterion, the activity of the presented solution can be reduced. In this paper, some new methods have been proposed to analyse the resources usage and the criteria to minimize their consumption. Thus, it can be concluded that these new schemes use fewer resources such as bandwidth, computational load and energy than the classical ones. The ideas presented in this work have been applied to an NCS for mobile robots as a practical approach. The navigation problem was solved using this new control paradigm and it has been compared with a classical discrete-time solution. Finally, the experimental results have demonstrated the stability and the efficiency in the resources usage of the proposed event-based control architecture if it is compared with classical control schemes.

In this work, the effects of the delays, the packet dropouts and the packet disorders in the network have not been analysed. To avoid these undesirable effects that the network produces in the proposed control system a free RF channel is selected to communicate the robots and the controller. This reduces the interference that other agents using the same frequency can produce. To improve the proposed system, a logic-like trigger [49] or other similar ideas could be applied in a simple way. To apply these methods, two main aspects must be previously developed. First, it is necessary to find a stochastic model of the mobile robots. Then, an exhaustive analysis of the network with different levels of congestion must be performed. With this model, an estimation of the delay between the packets and the volume of the disordered packets can be obtained. Finally, once these models have been developed a network-based H∞ filtering using a logic jumping-like trigger could be applied.

As a future work, the proposed strategy and the ideas presented in this paper will be analysed in systems such as Unmanned Aerial Vehicles (UAVs), Autonomous Underwater Vehicles (AUVs) or Legged Mobile Robots (LMRs). Furthermore, new strategies to ensure the stability and the efficiency of these systems will be investigated.

## Figures and Tables

**Table 1:** Experiment 1: obstacles avoidance algorithm.

Architecture	rn	$v_{n}$ (cm/s)	${\bar{e}}_{\mathrm{OA}}$ (cm)
Discrete-time	1	12	-
Event-based	2	12	0.3

**Table 2:** Experiment 2: wall following algorithm.

Architecture	rn	$v_{n}$ (cm/s)	*w* (cm)	$v_{w}$ (1/s)	${\bar{e}}_{\mathrm{WF}}$ (cm)
Discrete-time	1	12	1.5	-	-
Event-based	2	12	1.5	6	0.3

**Table 3:** Algorithm activity by architecture, number of samples, number of events, ratio of events and event efficiency.

Algorithm	$N_{\mathrm{per}}$	$N_{\mathrm{eve}}$	$N_{N}$	$\eta_{N}$
Obstacles Avoidance	9544	3548	0.37	63%
Wall Following	9544	7889	0.83	17%

**Table 4:** Bandwidth efficiency.

Algorithm	$({\bar{\mathrm{BW}}}_{\mathrm{DL}}{)}_{\mathrm{dis}}$	$({\bar{\mathrm{BW}}}_{\mathrm{DL}}{)}_{\mathrm{eve}}$	$N_{\mathrm{BW}}$	$\eta_{\mathrm{BW}}$
Obstacles Avoidance	509 bps	189 bps	0.37	63%
Wall Following	509 bps	421 bps	0.83	17%

**Table 5:** Algorithm operations.

**a) OA Algorithm**				
**Architecture**	${\mathrm{no}}_{C}$**(ops)**	${\mathrm{no}}_{R}$**(ops)**	${\mathrm{no}}_{\mathrm{Pd}}$**(ops)**	${\mathrm{no}}_{\mathrm{Pe}}$**(ops)**
Discrete-time	11	4	5	-
Event-based	11	10	-	3
**b) WF Algorithm**				
**Architecture**	${\mathrm{no}}_{C}$**(ops)**	${\mathrm{no}}_{R}$**(ops)**	${\mathrm{no}}_{\mathrm{Pd}}$**(ops)**	${\mathrm{no}}_{\mathrm{Pe}}$**(ops)**
Discrete-time	5	4	5	-
Event-based	5	10	-	3

**Table 6:** Computational load efficiency.

Algorithm	$(\bar{\mathrm{CL}}{)}_{\mathrm{dis}}$	$(\bar{\mathrm{CL}}{)}_{\mathrm{eve}}$	$N_{\mathrm{CL}}$	$\eta_{\mathrm{CL}}$
Obstacles Avoidance	12,725 ops/min	9674 ops/min	0.76	24%
Wall Following	8907 ops/min	10,570 ops/min	1.19	−19%

**Table 7:** Energy efficiency.

Algorithm	${\left(\mathrm{ER}\right)}_{\mathrm{dis}}$	${\left(\mathrm{ER}\right)}_{\mathrm{eve}}$	$N_{\mathrm{ER}}$	$\eta_{\mathrm{ER}}$
Obstacles Avoidance	18.09%	12.48%	0.69	31%
Wall Following	19.18%	16.56%	0.86	14%

## Appendix A. Pseudocodes of Control Algorithms

In this appendix, the pseudocodes of the following algorithms are presented:

- Algorithm 1: Code for the discrete-time robot, which is used for Obstacle Avoidance and Wall Following navigation algorithms.
- Algorithm 2: Code for the event-based robot, which is used for Obstacle Avoidance and Wall Following navigation algorithms.
- Algorithm 3: Code for the Obstacle Avoidance navigation algorithm, which is used in both architectures (discrete-time and event-based).
- Algorithm 4: Code for the Wall Following navigation algorithm, which is used in both architectures (discrete-time and event-based).

**Algorithm A1** Pseudocode in the discrete-time robot
$\mathrm{assign}\;sl\leftarrow v_{n}$ $\mathrm{assign}\;sr\leftarrow v_{n}$ while(true)do { 1: assignleftwheelspeed←sl 2: assignrightwheelspeed←sr 3: measureobstaclefrontleftsensor→fl 4: measureobstaclefrontrightsensor→fr 5: measureobstaclelateralleftsensor→ll 6: measureobstaclelateralrightsensor→lr 7: measurebatterylevel→bl 8: transmitsensorinformation(rn,fl,fr,ll,lr,bl) 9: receivecontrolinformation(sl,sr) }

**Algorithm A2** Pseudocode in the event-based robot
$\mathrm{assign}\;sl\leftarrow v_{n}$ $\mathrm{assign}\;sr\leftarrow v_{n}$ while(true)do { 1: assignleftwheelspeed←sl 2: assignrightwheelspeed←sr 3: measureobstaclefrontleftsensor→fl 4: measureobstaclefrontrightsensor→fr 5: measureobstaclelateralleftsensor→ll 6: measureobstaclelateralrightsensor→lr 7: measurebatterylevel→bl 8: if(eventcondition==true) { event_code() } else { 9: $\mathrm{assign}\;sl\leftarrow v_{n}$ 10: $\mathrm{assign}\;sr\leftarrow v_{n}$ } } event_code() { 1: transmitsensorinformation(rn,fl,fr,ll,lr,bl) 2: receivecontrolinformation(sl,sr) 3: return() }

**Algorithm A3** Pseudocode of the OA algorithm in the controller
1: receiveobstaclesensorinformation(fl,fr,ll,lr) 2: $\mathrm{check}\;\mathrm{condition}\;C_{1}$ 3: $\mathrm{check}\;\mathrm{conditions}\;C_{2},C_{3},C4$ 4: $\mathrm{check}\;\mathrm{conditions}\;C_{5},C_{6},C7$ 5: $\mathrm{check}\;\mathrm{condition}\;C_{8}$ 6: $\mathrm{check}\;\mathrm{condition}\;C_{9}$ // only in the discrete-time scheme 7: calculateω 8: calculatev 9: transform(ω,v)tosl 10: transform(ω,v)tosr 11: transmitcontrolinformation(sl,sr)

**Algorithm A4** Pseudocode of the FW algorithm in the controller
1: receiveobstaclesensorinformation(fl,fr,ll,lr) 2: checkcondition(fl>0) 3: calculatesl 4: calculatesr 5: transmitcontrolinformation(sl,sr)
